# Understanding the public’s intention to adopt CRISPR-Cas9: the effect of beliefs, knowledge, and innovativeness

**DOI:** 10.1007/s00439-026-02822-9

**Published:** 2026-03-02

**Authors:** Jasmine Melamed, Sivia Barnoy

**Affiliations:** Department of Nursing Sciences, Tel Aviv University, Tel Aviv, Israel

**Keywords:** CRISPR-Cas9, Gene editing, Beliefs, Diffusion of innovations, Knowledge

## Abstract

CRISPR-Cas9 is a gene editing technology with wide-ranging medical potential and significant ethical implications. This study examined how personality traits, familiarity with CRISPR, knowledge about CRISPR, and beliefs concerning its applications are connected to the public’s willingness to adopt this technology, drawing on Rogers’ Diffusion of Innovations framework. A sample of 500 young adults aged 20–45 completed questionnaires assessing innovativeness as well as familiarity, knowledge, beliefs, and willingness to adopt CRISPR-Cas9 for therapeutic and non-therapeutic purposes. Results showed that only 24% of participants were familiar with CRISPR and that knowledge levels were generally low. Based on Rogers’ typology, 18.8% were identified as innovators, and 26.8% as early adopters, and only 3.2% as laggards. Beliefs were strongly associated with willingness to adopt CRISPR-Cas9 (*r* = .63, *p* < .001), moderating the weak associations of personality traits with adoption intentions. These findings suggest that beliefs are an important factor in CRISPR-Cas9 adoption and they appear to have more influence on willingness to adopt CRISPR-Cas9 than knowledge and innovativeness in this sample. Efforts to promote informed public discussion on CRISPR should be made, with an emphasis on ethical aspects alongside scientific information. This is critical for the responsible adoption of this technology.

**Supplementary Information:**

The online version contains supplementary material available at 10.1007/s00439-026-02822-9.

## Introduction

CRISPR-Cas9 (Clustered Regularly Interspaced Short Palindromic Repeats - Cas Nuclease 9), a groundbreaking genome editing technology, has rapidly transitioned from basic science to clinical application. CRISPR-Cas9 is a powerful tool that can change DNA. The technology uses a guide RNA molecule in complex with the Cas9 endonuclease (an enzyme that cuts DNA) to make site-specific double-strand breaks, often called the “molecular scissors” method. The CRISPR part guides Cas9 to the exact spot in the DNA, where Cas9 makes the cut. Once cut, it is possible to remove, add, or replace pieces of DNA (Pacesa et al. 2024). Derived from an adaptive bacterial immune system, this platform thus enables precise gene disruption or insertion, including editing or correcting gene mutations in somatic cells. This facilitates functional genomics studies, the generation of disease models, and the development of therapeutic strategies for genetic disorders. In the latter case, approved treatments using CRISPR-Cas9, such as for sickle cell disease and beta-thalassemia, reflect its growing clinical potential (Lino et al. 2018; Pacesa et al. 2024).

However, the technology also raises ethical, social, and regulatory concerns regarding its potential usage in germ cells, particularly for non-therapeutic applications (Ramos et al. 2023). In 2018, a Chinese physician used CRISPR technology to produce genetically edited twins. He was sentenced to three years in prison (Cyranoski 2020). In a recent interview he declared that he will continue human editing with CRISPR (Higgins 2026). This case highlights the importance of exploring public opinion and shaping ethical guidelines to ensure responsible governance of human genome editing.

The public view of CRISPR is shaped by personality traits together with interest, hope, skepticism, and concern. A study conducted in the Netherlands among the general public found that religiosity predicted skepticism towards human gene editing (Većkalov et al. 2023). Another study conducted among participants under the age of 26 reported that younger participants expressed more optimism and support of therapeutic uses, together with concerns about ethical uses and equity (He 2025).

Public views and beliefs also depend on the intended use of CRISPR. A systematic review showed higher public support for the use of CRISPR for treating or preventing severe medical conditions, compared to its application for non-medical purposes such as enhancing intelligence or physical appearance (Ramos et al. 2023). Consistently, a study among young adults in the U.S. found more acceptance of CRISPR use in somatic cells compared to germline cells. The participants also expressed more approval for application of CRISPR for treating diseases and improving quality of life (Jones et al. 2023).

The public’s attitudes to CRISPR are affected by their levels of genetic literacy, trust in science, and ethical perspectives. While deeper knowledge can increase acceptance, it can also intensify concerns about long-term societal and biological consequences (Van Dijke et al. 2021). Indeed, knowledge of the technology was found to be associated with more belief in the likelihood of its clinical implementation in the next 20 years (So et al. 2021). A Dutch study that examined public views on Human Germline Genome Editing (HGGE) found that the majority agreed with the use of HGGE but only about 8.5% agreed to use of the technology for enhancement (Houtman et al. 2023). A recent study conducted by the Pew Research Center among 4726 US residents found that participants with a higher level of self-rated genetic knowledge expressed greater support for genetic enhancement (Halstead et al. 2023). This shows that knowledge is important for the formation of informed opinions about scientific discoveries. Taken together, these studies indicate that public attitudes toward CRISPR are shaped by an interplay of personality traits, intended applications, and levels of knowledge, highlighting the importance of participatory inclusion of diverse and informed public perspectives in the development of ethical guidelines and governance frameworks for gene-editing technologies.

Rogers’ (1983) Diffusion of Innovations (DOI) theory provides a comprehensive framework for understanding how new ideas, technologies, and practices are communicated and adopted within a social system over time. The theory was used to understand public uptake and willingness to adopt different health innovations such as the uptake of pharmacogenomics (Fuks Nielsen and Moldrup 2007), physicians’ acceptance of noninvasive prenatal testing (Wei et al. 2020), and the public’s knowledge and awareness of medical genetics in developing countries (Asadollahi et al. 2020). The DOI theory suggests a multi-stage adoption process that includes knowledge, persuasion, and decision making, each of which is influenced by individual personality traits, social norms, and access to reliable information (Sanson-fisher 2004). Moreover, individuals adopt new technologies at different rates based on distinct personality traits and social tendencies.

According to the theory, people can be categorized into five groups regarding adoption of technologies: innovators (2.5%), early adopters (13.5%), early majority (34%), late majority (34%), and laggards (16%) (Sahin et al. 2006). Innovators are adventurous and eager to try new ideas, often taking risks with little concern for social norms. Early adopters are respected opinion leaders who are open to change and help trigger wider acceptance. The early majority adopt new ideas just before the average person and tend to be deliberate and cautious. The late majority adopt after the average person and are typically skeptical, often due to economic or social pressures. Laggards are the last to adopt, usually bound by tradition and resistant to change. These categories are instrumental in understanding the spread of innovation and have been widely applied in fields such as healthcare, education, and information systems (Rogers 1983).

The knowledge stage of the DOI adoption process involves initial exposure to the innovation. Research shows that openness to experiences correlates with higher levels of knowledge acquisition (Goldsmith and Foxall 2003). Individuals who are more open to innovation tend to seek information early and form foundational understandings that later inform attitudes (Rogers 1983). In contrast, those who hold more traditional values may be less engaged in knowledge acquisition, resulting in lower familiarity and reduced acceptance of the innovation (Oyelana et al. 2021). During the persuasion stage of adopting innovations, individuals form beliefs about the innovation based on cognitive evaluation and emotional response. Thus, positive beliefs about CRISPR are linked to its potential to treat severe illnesses and improve quality of life (Jones et al. 2023), whereas concerns often stem from the need for better understanding or ethical uncertainties (McCaughey et al. 2019). A systematic review suggested that public engagement can enhance knowledge, resulting in better perceptions about CRISPR (Delhove et al. 2020).

The final DOI stage, decision making, reflects individuals’ readiness to adopt or reject the innovation. This readiness is not solely a function of knowledge; rather, it is shaped by the interaction of knowledge and belief. Studies have shown that individuals who are better educated and scientifically oriented tend to support CRISPR, particularly for therapeutic usages (Delhove et al. 2020). However, studies also suggest that beliefs can mediate the impact of knowledge and personality traits on the decision to adopt an innovation (So et al. 2021). For example, individuals with scientific knowledge can still hold strong moral objections that may cause them to reject an innovation. Indeed, religious or conservative groups may oppose it regardless of knowledge due to ethical and religious concerns (Van Dijke et al. 2021).

Although public attitudes toward CRISPR-Cas9 have been previously studied, theoretical frameworks have rarely been applied to examine how innovativeness, knowledge, and beliefs jointly shape adoption intentions, particularly for reproductive applications. Addressing this gap, the present study applies Rogers’ Diffusion of Innovations theory to examine these relationships among young adults of reproductive age, for whom such technologies are most directly relevant. Thus, the present study examines the relationships between personality traits, knowledge, beliefs, and adoption intentions of CRISPR-Cas9 technology among young adults of reproductive age in Israel.

## Methods

### Research procedure

In the first stage, the questionnaires (on knowledge, beliefs, and willingness to use) developed for the study were distributed to 30 participants to assess their clarity and participant responsiveness. These participants were recruited through social media groups focused on medical technologies. These completed questionnaires were not included in the analysis. In the second stage, the questionnaires were distributed online to 500 participants via the survey company iPanel©. The company operates the largest online respondent panel in Israel, consisting of approximately 100,000 active members (http://ipanel.co.il/en/). Panel members receive modest incentives for participation in surveys, in accordance with standard panel practices. iPanel is a reliable company and its samples are representative of the Israeli population (Bodas and Peleg 2020). It is a member of the European Society for Opinion and Marketing Research (ESOMAR) and adheres to international data collection standards to ensure the highest data quality (Bowers 1998; Passingham et al. 2016). Such strategies include adding dummy questions to check respondent attentiveness and maintain data integrity, ongoing daily recruitment of new panel members, as expanding the panel and bringing in new participants to prevent various effects of panelist professionalization. In addition, it incorporates restrictions on survey frequency per participant and mechanisms for identifying inconsistent or invalid responses. The questionnaires were sent randomly to eligible Israeli young adults by email or SMS using the Qualtrics survey platform.

Before completing the questionnaires, participants received an explanation about CRIPSR-Cas9 that included a brief description of CRISPR technology and its potential usage and applications. The explanation provided was: “CRISPR is a gene-editing technology that can potentially prevent and cure currently untreatable diseases. In addition, CRISPR can be used to make genetic changes and enhancements. When this technology is applied in germline cells, the genetic change is passed on to future generations. In most cases, it is not yet possible to know how such changes might affect other areas of health, including potential risks such as cancer or other diseases. While it offers great medical promise, it also raises important ethical and social questions, such as whether it should be used only for therapeutic purposes (treating diseases) or also for human enhancement (e.g., improving physical or cognitive traits)”.

The distribution took place after obtaining approval from Tel Aviv University ethics committee (#0004129-1) which gave an exemption for signing an informed consent form since accessing the questionnaires after a screened explanation of the study online was considered informed consent.

### Research tools

The research tools included the following five questionnaires, presented to participants in sequence. The first was a socio-demographic questionnaire, which included a yes/no screening question at the end assessing participants’ familiarity with CRISPR. Participants who indicated familiarity with CRISPR were directed to the knowledge questionnaire, followed by questionnaires assessing beliefs, willingness to use CRISPR-Cas9, and personality traits. Participants who reported no prior familiarity with the technology were directed to the beliefs questionnaire, followed by questionnaires assessing beliefs, willingness to use CRISPR-Cas9, and personality traits.

*Socio-demographic details* Data were collected regarding gender, age, religiosity, education level, marital status, economic status, and reported health status (see Table 1).

**Table 1:** Socio-demographic background variables of the sample (*n* = 500)

Variable	Category	Respondents *N* (%)
Gender (*N* = 500)	Female	260 (52%)
	Male	240 (48%)
Age (years)	20–25	84 (17%)
	26–30	97 (19%)
	31–35	114 (23%)
	36–40	133 (27%)
	41–45	72 (14%)
Level of Religiosity	Secular	220 (44%)
	Traditional	175 (35%)
	Religious	55 (11%)
	Ultra-Orthodox	50 (10%)
Education	Elementary or lower	5 (1%)
	Secondary	119 (24%)
	Post-secondary	111 (22%)
	Bachelor’s degree	173 (35%)
	Master’s degree or higher	92 (18%)
Marital status	Single	156 (31%)
	Married/have a partner	328 (66%)
	Divorced	16 (3%)
Region of residence	North	47 (9.4%)
	Haifa	63 (12.6%)
	Center	159 (31.8%)
	Tel Aviv	112 (22.4%)
	Jerusalem	44 (8.8%)
	South	75 (15%)
Health status	Generally healthy	488 (98%)
	With chronic illness	12 (2%)
Economic status	Below average	226 (45%)
	Average or above	243 (49%)
	No response	31 (6%)

*CRISPR Knowledge Questionnaire* (see Appendix 1): This questionnaire was developed for the present study based on the literature on genetic literacy and public understanding of CRISPR (Jones et al. 2023; McCaughey et al. 2019; Yang and Hobbs 2020). Items covered basic concepts (e.g., mechanisms of CRISPR-Cas9), potential applications (therapeutic vs. enhancement), and associated risks. Before completing the questionnaire, participants were asked whether they are familiar with CRISPR technology. Those who answered “Yes” proceeded to fill out the questionnaire, while those who answered “No” were automatically directed to the next questionnaire. The questionnaire included 16 items, with each item having three response options: “True”, “False”, and “Don’t Know”. The “Don’t Know” option was intentionally included to reduce guessing and minimize response bias. Participant scores were calculated based on the number of correct answers; Cronbach’s α = 0.83.

*Beliefs Questionnaire* (see Appendix 1): This questionnaire was constructed for the present study to examine participants’ beliefs regarding the use of CRISPR-Cas9 technology for various applications, such as disease treatments/prevention and enhancement. The questionnaire was based on existing questionnaires previously used in genetic editing research (Beckman et al., 2019; Taguchi et al. 2019), and aimed to determine whether participants perceive CRISPR-Cas9 gene editing technologies as positive or negative. The questionnaire included 12 statements, with responses given on a 5-point scale ranging from 1 = strongly disagree to 5 = strongly agree. Items 5–6 and 8–10 were reversed-scored due to their opposite wording. The scores were calculated as the average of all responses, with higher scores indicating more positive beliefs. The internal reliability of the scale was α = 0.702.

Q*uestionnaire on Willingness to Use CRISPR*-*Cas9* (see Appendix 1): This questionnaire was developed for the present study to measure willingness to use CRISPR-Cas9 for different purposes (the “decision making” stage in Rogers’ DOI theory) based on the literature that addressed similar aspects (Critchley et al. 2019; Jedwab et al. 2020; Jones et al. 2023). Before completing the questionnaire, participants received an explanation about CRIPSR-Cas9. In addition, they were given definitions of the ages mentioned in the questionnaire (e.g., after birth = 0–1 years, children and adolescents = 1–25 years, adulthood = 25+), as well as a classification of disease types and severity levels (Lazarin et al. 2014). These explanations aimed to ensure full understanding of the questionnaire. The questionnaire included 18 items examining the degree to which participants are willing to adopt or reject CRISPR-Cas9 technology for therapeutic purposes (11 items) and non-therapeutic purposes (7 items; items 2, 4–7, 17–18, see Appendix 1). Responses were rated on a 5-point Likert scale (1 = strongly disagree, 5 = strongly agree). The scores were calculated as the mean average of responses to the 18 items. Higher scores indicated greater willingness to adopt the technology. The internal reliability of the scale was α = 0.95.

*Personality Traits Questionnaire (Individual Innovativeness Scale – IIs)* (see Appendix 1): This questionnaire measures individuals’ attitudes toward innovation in the context of technology adoption. Developed by Hurt et al. (1977), it assesses personality traits related to innovativeness and allows classification into Rogers’ (1983) five adopter categories. The scale includes items assessing perceptions of new ideas, self-assessed leadership ability, attitudes toward uncertainty, and openness to new inventions (Hurt, et al.,^1977^). The questionnaire was translated into Hebrew using the translation-back-translation method and consisted of 20 items. To ensure consistency across all study questionnaires, all items were rated on a 5-point Likert scale ranging from 1 = strongly disagree to 5 = strongly agree. Following the original scoring procedure, responses to 12 positively keyed items (S₁) and 8 negatively keyed items (S₂) were summed, and an Innovation Index (II₅) was calculated as II = 42 + S₂ − S₁. Because the original adopter-category cutoffs were developed using a 7-point response format, direct comparison required converting our 5-point-based index to the 7-point metric. To ensure conceptual equivalence with the original classification system, we linearly rescaled the index scores from the range of the 5-point format to the corresponding range of the 7-point format. This min–max transformation preserves the relative position of each participant within the scale distribution while adjusting the metric to match the original scoring framework. The resulting rescaled index (II₇) that allowed the calcification into Rogers’ adopter categories using established cutoffs: Innovators (> 80), Early Adopters (69–80), Early Majority (57–68), Late Majority (46–56), and Laggards (< 46). The internal reliability of the scale in the present study was satisfactory (Cronbach’s α = 0.83).

### Participants

The study included 500 participants (52% women) who were Hebrew-speaking and aged 20–45. The sample was intentionally restricted to young adults aged 20–45, as this group is most directly relevant to the reproductive applications of CRISPR-Cas9, including potential prenatal genetic interventions. Accordingly, the aim of the study was to examine beliefs and willingness to adopt CRISPR-Cas9 within a population for whom these technologies are likely to be personally relevant.

Data were collected between July-August 2022 by iPanel©, via the Qualtrics survey platform. The initial sample size was calculated using the G*Power software, which determined that at least 138 participants were required to achieve a test power of 0.95 and a reliability of 0.05. In consultation with the iPanel© company, a decision was made to expand the sample to 500 participants to more accurately represent the general public and reflect the demographic and socio-economic diversity of the general population, reduce sampling error, and increase the precision of subgroup analyses.

### Data analysis

Data analysis was performed using IBM^®^ SPSS^®^ Statistics version 28. Descriptive statistics (means, standard deviations, percentages, and distributions) were calculated to summarize the sample characteristics. Associations between continuous variables were assessed using Pearson correlation coefficients. Group differences in willingness to adopt the CRISPR technology were analyzed using independent samples t-tests and one-way analysis of variance (ANOVA). Williams’ test was applied to compare the relative strength of correlations. Finally, for the moderation analysis, all variables were mean-centered, and moderation effects were tested using interaction analyses conducted with PROCESS Model 1.

## Results

The sample included 500 participants, including 240 men and 260 women aged 20 to 45 years. The majority identified as secular (44%) or traditional (35%) and as married/having a partner (66%). 53% had an academic education, with 33% holding a bachelor’s degree and 18% a master’s degree. About 55% of participants were living in the center of the country (31.8%) or the Tel Aviv area (22.4%), and 22% in the northern region and Haifa and 23% in Jerusalem and the southern region. This distribution of respondents across regions broadly reflects the population distribution in Israel. Most of the participants (98%) reported being in good health with no pre-existing medical conditions. Only 24% (*n* = 122) claimed to be familiar with CRISPR technology. Within this group, most (59%) assessed their knowledge level as moderate, a third (33%) as low, and only 7% as high. See Tables 1 and 2 presents the descriptive statistics of study variables.

**Table 2:** Descriptive statistics of the study variables#

Variable			
	M SD	Minimum	Maximum
Knowledge	6.68 3.11	0.00	13.00
Beliefs	2.95 0.54	1.42	4.83
Personality innovativeness	68.31 13.45	34.00	116.50
Willingness to adopt CRISPR	2.77 0.86	1.00	5.00
Willingness to adopt for Cure	3.00 0.96	1.00	5.00
Willingness to adopt for Enhancement	2.42 0.86	1.00	5.00

# Knowledge: *n* = 122; all other variables: *n* = 500

Based on Rogers’ (1983) DOI personality classification, 94 participants (18.8%) were identified as innovators, 134 (26.8%) as early adopters, 174 (34.8%) as early majority, 82 (16.4%) as late majority, and 16 (3.2%) as laggards. Correlation analyses revealed a weak but significant relationship between participants’ knowledge and beliefs (*n* = 500; *r* = .20, *p* < .05) but the association between their personality traits and knowledge was not significant (*n* = 122; *r*=.15). By contrast, a strong positive association was found between participants’ beliefs and their willingness to adopt the technology (*r* = .63, *p*<.001) (see Table 3). To further examine the latter relationship, two additional correlations were performed: (1) between participants’ beliefs and their willingness to use the technology for treating genetic diseases (*r*=.53; *p*<.001), and (2) between their beliefs and their willingness to use the technology for genetic enhancement (*r*=.60; *p*<.001). A Williams’ test comparing overlapping dependent correlations indicated a statistically significant difference (t(497) = 3.03 *p*=.001) (Neill and Dunn 1975), showing that the correlation between participants’ beliefs and their willingness to use CRISPR technology for enhancement purposes was significantly stronger than the corresponding correlation for treatment purposes.

**Table 3:** Correlations between the research variables#

Variable	1	2	3
1. Personality traits	–		
2. Knowledge	0.15	–	
3. Beliefs	0.04	0.20*	–
4. Willingness to adopt CRISPR	0.10*	0.006	0.63***

*p < .05*, **p < .01, ***p < .001

#Knowledge: *n* = 122; all other variables: *n* = 500

An independent samples t-test was performed to assess differences between men and women’s willingness to adopt CRISPR-Cas9 and their beliefs about it. The results indicated that men demonstrated significantly higher willingness to adopt the technology compared to women (*p* = .025), with a small to moderate effect size (Cohen’s d=0.32). However, gender differences in beliefs about CRISPR were not statistically significant (*p*=.058) (Table 4).

**Table 4:** Independent samples t-test for gender differences in willingness to adopt CRISPR-Cas9 and beliefs (*n* = 500)

Variable	Men *(N* = 240)	Women *(N* = 260)	t	*p*
	M SD	M SD		
Willingness to adopt CRISPR	2.86 0.89	2.69 0.82	2.25	0.025*
Beliefs	2.87 0.60	2.77 0.55	1.91	0.058

To explore differences by religiosity, a one-way ANOVA was conducted. The ANOVA indicated significant differences across religiosity groups, F(3, 496) = 7.11, *p* < .001, η² = 0.041, indicating a small effect size. As shown in Table 5, secular and traditional participants (M = 2.86, SD = 0.86) reported higher levels of willingness to adopt CRISPR-Cas9 compared to religious and ultra-Orthodox participants (M = 2.46, SD = 0.79). The difference between these groups was statistically significant, t = 4.35, *p* < .01.

**Table 5:** Means, standard deviations, and one-way ANOVA for willingness to adopt CRISPR-Cas9 by level of religiosity

Group	*N*	M	SD	F(3, 496)	*p*	η²
Secular	220	2.88	0.85			
Traditional	175	2.83	0.85			
Religious	55	2.57	0.71			
Ultra-Orthodox	50	2.33	0.86			
ANOVA				7.11	< 0.001***	0.041

**Fig. 1:**
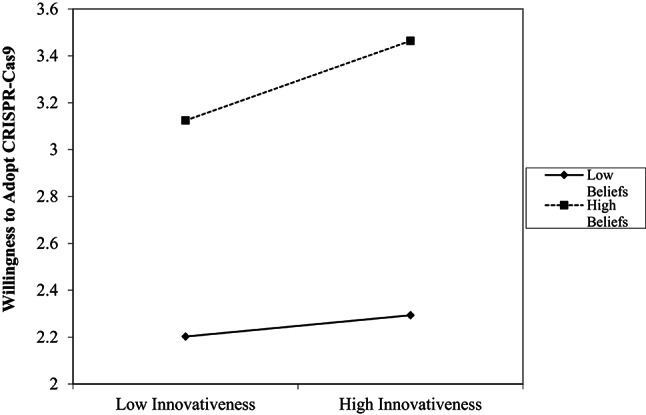
The moderating effect of belief levels on the relationship between types of adopters and the decision to adopt CRISPR-Cas9 technology

Finally, to further explore the relationships between the variables, a moderation analysis was conducted. Specifically, the interaction between participants’ personality traits (innovativeness) and their beliefs about CRISPR-Cas9 in predicting willingness to adopt the technology was examined. The regression analysis revealed significant main effects of beliefs, *B* = 0.91, *p* < .001, and innovativeness, *B* = 0.01, *p* < .001, on readiness to adopt CRISPR-Cas9. These effects were qualified by a significant interaction between innovativeness and beliefs, *B* = 0.01, *p* = .021. Simple slope inspection indicated that innovativeness was more strongly associated with readiness among individuals with high beliefs, whereas this association was weak among individuals with low beliefs (Fig. 1).

## Discussion

This study offers important insights into the public’s familiarity with and the CRISPR-Cas9 technology and beliefs it about among a large young adult population. By integrating the DOI theory (Rogers 1983) with empirical data on knowledge, beliefs, and adoption intentions, this study extends existing research, clarifying the mechanisms underlying public willingness to adopt CRISPR-Cas9 among individuals of fertility age. In addition, the current findings are particularly important given that systematic, nationally representative research on public attitudes toward human gene editing is scarce, especially outside the United States, resulting in a limited understanding of public perspectives at the global level (Howell et al. 2020).

The present sample consisted of relatively young, generally healthy Israeli men and women. This profile reflects an intentional design choice rather than a sampling limitation, as participants were recruited specifically within the fertility age range, which is particularly relevant to the research questions regarding willingness to use CRISPR-Cas9 for fetuses and after birth. Indeed, calls for inclusive public engagement emphasize the importance of capturing perspectives from populations most likely to be directly affected by human gene-editing applications (Howell et al. 2020), such as individuals of reproductive age. At the same time, individuals within this age group may differ from the broader Israeli population in terms of health status, life experience, and engagement with research participation. Hence, the findings are best interpreted in the context of individuals within the fertility age range, who represent the study’s target population.

Despite the media attention, familiarity with CRISPR-Cas9 remains low, with only 24% of participants reporting awareness of the technology. Even among those familiar with it, self-reported knowledge was generally moderate to low. These findings are surprising in light of the high proportion of innovators and early adopters in our sample. According to the DOI theory (Rogers 1983), individuals with these adopter profiles are typically more inclined to seek information and acquire knowledge about innovations. However, these findings are consistent with prior research that failed to find correlations between knowledge, awareness of scientific innovations, and approval of genetic editing (So et al. 2021). Indeed, studies indicate that public knowledge about CRISPR remains limited, and much of the existing research on public attitudes toward the technology has been conducted in the United States, with comparatively little data from other countries (Baik et al. 2022). In Israel, the absence of local studies and the limited Hebrew language content may further restrict publicly accessible knowledge. Since public resistance to gene editing is connected to the need for more information (McCaughey et al. 2019), the low familiarity with CRISPR-Cas9 reported in the present study may also have influenced its acceptance.

The observed lack of association between personality traits and knowledge was surprising, particularly given the large proportion of innovators and early adopters in the sample, and appears inconsistent with the DOI theory, which predicts a positive correlation between innovativeness and the tendency to seek out knowledge. Accordingly, innovators and early adopters are the first to seek knowledge about new technologies (Rogers 1983). This discrepancy between the current findings and the DOI theory may stem from the scientific complexity of molecular and genetic engineering concepts, as well as the perceived ambiguity of CRISPR. These factors may be barriers to gaining knowledge for the broader public even among individuals who are open to innovation. Consequently, it has been suggested that the scientific community should act as knowledge facilitators and disseminate knowledge about CRISPR to the public (Baik et al. 2022).

According to Rogers’ (1983) DOI theory, social and cultural factors may affect the rate of knowledge adoption even given a personality-based tendency toward innovations. This study’s sample revealed an atypically high proportion of “innovators” and “early majority” individuals (45.6%), and an underrepresentation of “laggards” (3.2%). The high proportion of innovators observed in our study may reflect the innovation oriented culture that characterizes Israeli society (Yeshua-Katz & Treister, 2021). At the same time the overrepresentation of innovators and early majority adopters could also reflect broader societal tendencies, where individuals await more evidence and consensus before embracing novel biotechnologies (unlike innovators and early adopters). In this context, the cultural norms and social conservatism prevalent in Israel may act as inhibiting factors, particularly when information about the technology is not available in Hebrew. This situation highlights the importance of examining the roles of religion, culture, and the perceived complexity of CRISPR in understanding the relationship between personality traits and levels of knowledge.

One of the most notable findings of this study is the central role of beliefs in shaping willingness to adopt CRISPR-Cas9, with a strong positive correlation found between these two factors. These results are consistent with broader calls to move beyond knowledge-deficit approaches toward capturing public values and perspectives as key inputs in gene-editing governance (Howell et al. 2020). As Grayling (2011) argues, individuals often form beliefs first and only then seek evidence to support them (Grayling 2011). This suggests that affective and cognitive evaluations such as perceived risks, benefits, and moral acceptability may contribute to beliefs regarding gene editing. This finding also aligns with Rogers’ (1983) theory, which emphasizes that the persuasive stage of technology adoption depends on forming favorable beliefs about an innovation’s relative advantages. Similarly, Scheufele et al. (2021) found that positive beliefs and trust in scientists strongly predicted willingness to adopt innovative technologies such as CRISPR.

The moderation analysis findings further clarified the central role of beliefs. While personality traits alone were weak predictors of adoption, their influence became more pronounced when combined with beliefs. This moderation underscores the complexity of technology adoption decisions, which depend not only on individual predispositions but also on contextual and cognitive evaluations of the technology. Chen and Zhang (2022) also reported a moderating effect of beliefs on the relationship between knowledge, attitudes, risk-benefit perception, and acceptance of human gene editing, supporting their pivotal role in adoption decisions.

Another unexpected finding was the significantly stronger correlation between beliefs and willingness to use CRISPR for enhancement purposes as compared to treatment of genetic diseases, especially given the ethical concerns often associated with the former. However, a previous study did report that only half of its sample disapproved usage of genetic gene editing technologies for enhancement (Jedwab et al. 2020). Our finding may be consistent with a possible shift toward greater acceptance of enhancement technologies among the public, and future research should examine the role of cultural portrayals of genetic “optimization” and human advancement. The finding may also stem from the nature of our sample, which consisted of young adults who are generally healthy. As such, disease treatment may be less relevant to this population, whereas enhancement may be more aligned with their age and life stage.

The gender-based differences, where men showed significantly higher willingness to adopt CRISPR-Cas9 than women, aligns with previous literature showing more openness to adopt new technologies among men (Weisberg et al. 2017). Interestingly, no gender differences emerged in beliefs, suggesting that while beliefs may be similar, behavioral intentions diverge between the genders. Religiosity also emerged as a significant factor. Secular and traditional participants expressed more willingness to adopt CRISPR than their religious and ultra-Orthodox counterparts. This is consistent with prior studies showing that religious people are less inclined to adopt new technologies, especially those perceived as “playing God” or altering natural life (Jedwab et al. 2020; McCaughey et al. 2019).

In conclusion, this study points to an interplay of personality, knowledge, and beliefs in the intention to adopt CRISPR-Cas9 technology. Our findings suggest that public beliefs are associated with the intention to adopt gene editing. Given the rapidly evolving field of human gene editing, continued well-designed empirical research on public attitudes and beliefs, attentive to social and cultural factors, remains important for informing public dialogue and policy discussions. As Howell et al. (2020) argue, without sustained and representative research on public opinion, efforts to foster meaningful public discourse and evidence-informed policymaking in this domain may be limited (Howell et al. 2020). Accordingly, policymakers and science communicators may benefit from considering inclusive approaches that take emotional and cultural factors into account alongside scientific information. Beyond conveying technical facts, outreach efforts that acknowledge public values and concerns could facilitate more constructive engagement with CRISPR and related technologies. It has been suggested that such approaches may help support informed dialogue and contribute to ongoing discussions about the regulation of emerging biotechnologies. (Lino et al. 2018; Ramos et al. 2023).

### Implications

Enhancing public understanding of CRISPR, in particular its medical benefits and risks, could improve informed decision-making. Since beliefs may form prior to knowledge and serve as a powerful driver of adoption, efforts to shape public discourse should prioritize clarity, transparency, and ethical engagement. Future research should further investigate factors related to CRISPR adoption intentions, including concerns and ethical issues, perhaps through qualitative methods, to unpack the underlying reasoning.

### Limitations

This study has several limitations. Participants were recruited via a commercial survey panel, which may introduce self-selection bias, as respondents willing to complete multiple questionnaires may differ from the general population in motivation or interest in health-related research. In addition, participants in our study received incentives through the survey company. While such incentives are standard practice in online survey research and help improve recruitment and completion rates, they may introduce potential response bias. For example, some participants may be primarily motivated by compensation rather than intrinsic interest, which could affect attentiveness or response quality. However, the survey company adheres to ESOMAR guidelines (Passingham et al. 2016), which support the quality and broad representativeness of the sample. The findings are best interpreted in relation to individuals within the fertility age range. Also, the sample was restricted to Hebrew-speaking young adults in Israel, limiting generalizability across cultures, languages, and age groups. Though internally reliable, the beliefs, knowledge, and willingness to adopt CRISPR-Cas9 technology questionnaires were newly developed for the present study, and not externally validated. As a cross-sectional survey, the study cannot establish causality, and responses may have been influenced by the brief explanatory text provided before the questionnaires. Finally, other potential influences such as trust in science, political orientation, or media exposure were not directly assessed.

## Supplementary Information

Supplementary Material 1.

## Data Availability

Dats will be avialable by request.
