# Supplementary material for: Understanding the public’s intention to adopt CRISPR-Cas9: the effect of beliefs, knowledge, and innovativeness
Source: Hum Genet. 2026 Mar 2;145(1):28. doi: 10.1007/s00439-026-02822-9 (PMC12953347; doi:10.1007/s00439-026-02822-9)
Supplement: Supplementary file 1 — Supplementary Material 1. [file 439_2026_2822_MOESM1_ESM.docx]

**Appendix 1**

Dear Participant

This study focuses on CRISPR technology.
CRISPR is a new technology that allows precise editing of the DNA, the molecule that carries the genetic instructions of our bodies. In following questionnaires, you will be asked about your knowledge, and what you think and feel about the topic. Please read each item carefully, and answer according to your personal feeling and knowledge.

Thank you for your cooperation.

## CRISPR Knowledge Questionnaire

| Statement | True | False | Don't Know |
| --- | --- | --- | --- |
| 1. CRISPR is a technology that is remains under active research with most of its medical applications tested in clinical trials | X |  |  |
| 1. CRISPR is a technology that deals with genetic editing | X |  |  |
| 1. CRISPR is a technology that helps with fertility |  | X |  |
| 1. CRISPR use is possible only in reproductive cells (and not applicable to babies) |  | X |  |
| 1. CRISPR is already used in medical treatments such as in Thalassemia | X |  |  |
| 1. CRISPR use has the potential to cure or prevent incurable diseases | X |  |  |
| 1. CRISPR is a new genetic technology that has caused a lot of ethical concerns and debates around the world. | X |  |  |
| 1. CRISPR can be used to prevent natural aging |  | X |  |
| 1. CRISPR was discovered through bacterial research | X |  |  |
| 1. CRISPR use has the potential to change physical traits (e.g., eye color) at the embryonic stage | X |  |  |
| 1. When CRISPR is used in an embryo, the change is passed on to future generations | X |  |  |
| 1. CRISPR is accessible, cheap, and easy to operate | X |  |  |
| 1. CRISPR can be used to extend life expectancy |  | X |  |
| 1. CRISPR was discovered in the past year |  | X |  |
| 1. CRISPR can already be used to change a person's IQ |  | X |  |
| 1. CRISPR can be used in agriculture (animals, plants, and vegetables) to improve genetic traits | X |  |  |

## Beliefs Questionnaire:

CRISPR technology that allows precise editing of the DNA. With this technology, we can better understand how genes affect the body and potentially use it to treat or prevent various diseases or for genetic enhancement.

The following questionnaire refer to your beliefs about CRISPR genetic editing technology applications, please mark your personal belief on each item:

| **I believe that using CRISPR technology:** | 1  Strongly Disagree | 2  Disagree | 3  Moderately Agree | 4  Agree | 5  Strongly Agree |
| --- | --- | --- | --- | --- | --- |
| 1. for genetic enhancement (changing physical traits, intelligence, skin color, etc.) can lead to desirable result |  |  |  |  |  |
| 1. for disease prevention is desirable |  |  |  |  |  |
| 1. should not be restricted and should be approved for all uses |  |  |  |  |  |
| 1. has the potential to contribute positively to human progress |  |  |  |  |  |
| 1. should be confined, at this stage, to research purposes only |  |  |  |  |  |
| 1. can have harmful effects on future generations |  |  |  |  |  |
| 1. can be helpful for other people |  |  |  |  |  |
| 1. raises important ethical concerns |  |  |  |  |  |
| 1. goes conflicts with my personal values |  |  |  |  |  |
| 1. could contribute to greater social inequality |  |  |  |  |  |
| 1. will not dramatically change humanity |  |  |  |  |  |
| 1. in reproduction could improve future generations |  |  |  |  |  |

**Willingness to Use CRISPR Questionnaire**

CRISPR is a gene-editing technology that can prevent and cure incurable diseases. Additionally, it can be used for genetic changes and enhancements. When this technology is used in germline (reproductive) cells, the genetic change can be passed on to future generations, and in most cases, it is impossible to know how it may affect other areas, including the risk of cancer or other diseases.

The items below use the following grouping of ages and disease severity;

**After birth**: 0–1 years; **Childhood and adolescence**: 1–25 years; **Adulthood**: 25+ years

**Mild diseases** – Diseases that mildly affect life and quality of life and do not shorten life expectancy. These are usually treatable and manageable.
**Moderate diseases** – Diseases that are incurable but do not shorten life expectancy. They moderately affect quality of life and are usually manageable with symptom-relief treatments.
**Severe diseases** – Diseases that may shorten life expectancy and severely affect life. No curative treatment exists, only symptom relief.

The following questions refer to your level of agreement with the use of CRISPR genetic editing for the purposes listed below:

|  | 1  Strongly Disagree | 2  Disagree | 3  Moderately Agree | 4  Agree | 5  Strongly Agree |
| --- | --- | --- | --- | --- | --- |
| 1. Treating/preventing severe diseases in your children |  |  |  |  |  |
| 1. Enhancing traits in your children |  |  |  |  |  |
| 1. Treating a disease that may develop in the future in yourself |  |  |  |  |  |
| 1. Determining physical appearance in your embryo (eye/hair/skin color, height) (before birth) |  |  |  |  |  |
| 1. Determining intelligence level of your embryo (before birth) |  |  |  |  |  |
| 1. Determining strength or athletic abilities in your embryo (before birth( |  |  |  |  |  |
| 1. If no one will know about the use |  |  |  |  |  |
| 1. Preventing/treating a mild disease appearing after birth |  |  |  |  |  |
| 1. Preventing/treating a moderate disease appearing after birth |  |  |  |  |  |
| 1. Preventing/treating a severe disease appearing after birth |  |  |  |  |  |
| 1. Preventing/treating a mild disease appearing in childhood |  |  |  |  |  |
| 1. Preventing/treating a moderate disease appearing in childhood |  |  |  |  |  |
| 1. Preventing/treating a severe disease appearing in childhood |  |  |  |  |  |
| 1. Preventing/treating a mild disease appearing in adulthood |  |  |  |  |  |
| 1. Preventing/treating a moderate disease appearing in adulthood |  |  |  |  |  |
| 1. Preventing/treating a severe disease appearing in adulthood |  |  |  |  |  |
| 17. Correction of a physical defect/malformation in the fetus (before birth) |  |  |  |  |  |
| 18. A change that will transfer to future generations |  |  |  |  |  |

**Personality Traits Questionnaire (Hurt, Joseph & Cook, 1977)**

People respond to their environment in different ways. The following statements refer to some of these responses.
Please rate your level of agreement with each statement by choosing one of the following options:

**1 – Strongly Disagree**
**2 – Disagree**
**3 – Moderately Agree (Neutral)**
**4 – Agree**
**5 – Strongly Agree**

|  | 1  Strongly Disagree | 2  Disagree | 3  Moderately Agree | 4  Agree | 5  Strongly Agree |
| --- | --- | --- | --- | --- | --- |
| 1. My peers often ask me for advice or information |  |  |  |  |  |
| 1. I enjoy trying new ideas |  |  |  |  |  |
| 1. I seek out new ways to do things |  |  |  |  |  |
| 1. I am generally cautious about accepting new ideas |  |  |  |  |  |
| 1. I frequently improvise methods for solving a problem when an answer is not apparent |  |  |  |  |  |
| 1. I am suspicious of new inventions and new ways of thinking |  |  |  |  |  |
| 1. I rarely trust new ideas until I can see whether the vast majority of people around me accept them |  |  |  |  |  |
| 1. I feel that I am an influential member of my peer group |  |  |  |  |  |
| 1. I consider myself to be creative and original in my thinking and behavior |  |  |  |  |  |
| 1. I am aware that I am usually one of the last people in my group to accept something new |  |  |  |  |  |
| 1. I am an inventive kind of person |  |  |  |  |  |
| 1. I enjoy taking part in the leadership responsibilities of the groups I belong to |  |  |  |  |  |
| 1. I am reluctant about adopting new ways of doing things until I see them working for people around me |  |  |  |  |  |
| 1. I find it stimulating to be original in my thinking and behavior |  |  |  |  |  |
| 1. I tend to feel that the old way of living and doing things is the best way |  |  |  |  |  |
| 1. I am challenged by ambiguities and unresolved problems |  |  |  |  |  |
| 1. I must see other people using new inventions before I will consider them |  |  |  |  |  |
| 1. I am receptive to new ideas |  |  |  |  |  |
| 1. I am challenged by unanswered questions |  |  |  |  |  |
| 1. I often find myself skeptical of new ideas |  |  |  |  |  |
